# Case of Malformation of the Heart

**Published:** 1805-04-01

**Authors:** 


### ADDENDA.

### *Case of Malformation of the Heart, by Mr. Ring, vide p.* 120.

### EXPLANATION OF THE PLATE.

ANTERIOR VIEW.

*a.* The superior cava
*b.* The aorta
*c.* The right auricle
*d.* The opening of the pulmonary artery
*e.** The opening of the aorta
*f.* The opening of the right auricle
*g.* The right ventricle
*h.* The pulmonary artery
*i.* The left aurcle
*k.* The opening of the aorta
*l.* The opening of the left auricle
*m.* The left ventricle

POSTERIOR VIEW.

*n.** The superior cava
*o* The pulmonary artery
*p.* The aorta
*q.** The inferior cava
*r.* The left auricle
*s.* The left ventricle
*t.* The superior cava
*u.* The right auricle
*v.* The right ventricle

Those marked with a * are deviations from the natural structure; *d. e. f. k.* and *l.* refer to the foramina, opposite to which they are placed.

